# FDSNet: dynamic multimodal fusion stage selection for autonomous driving via feature disagreement scoring

**DOI:** 10.1038/s41598-025-25693-y

**Published:** 2025-12-19

**Authors:** Asaad Mohammed, Hosny M. Ibrahim, Nagwa M. Omar

**Affiliations:** Information Technology Department, Faculty of Computers and Information, Assiut University, Assiut, 71515 Egypt

**Keywords:** Engineering, Mathematics and computing

## Abstract

Robust and efficient 3D perception is critical for autonomous vehicles operating in complex environments. Multi-sensor fusion, such as Camera+LiDAR, Camera+Radar, or all three modalities, significantly enhances scene understanding, However, most existing frameworks fuse data at a fixed stage, categorized as early fusion (raw data level), mid fusion (intermediate feature level), or late fusion (detection output level), neglecting semantic consistency across modalities. This static strategy may result in performance degradation or unnecessary computation under sensor misalignment or noise. In this work, we propose FDSNet (Feature Disagreement Score Network), a dynamic fusion framework that adaptively selects the fusion stage based on measured semantic consistency across sensor modalities. Each sensor stream (Camera, LiDAR, and Radar) independently extracts mid-level features, which are then transformed into a common Bird’s Eye View (BEV) representation, ensuring spatial alignment across modalities. To assess agreement, a Feature Disagreement Score (FDS) is computed at each BEV location by measuring statistical deviation across modality features. These local scores are aggregated into a global FDS value, which is compared against threshold to determine the fusion strategy. A low FDS, indicating strong semantic consistency across modalities, triggers mid-level fusion for computational efficiency, whereas a high FDS value activates late fusion to preserve detection robustness under cross-modal disagreement. We evaluate FDSNet on the nuScenes dataset across multiple configurations: Camera+Radar, Camera+LiDAR, and Camera+Radar+LiDAR. Experimental results demonstrate that FDSNet achieves consistent improvements over recent multimodal baselines, with gains of up to +3.0% in NDS and +2.6% in mAP on the validation set, and +2.1% in NDS and +1.6% in mAP on the test set, highlighting that dynamic stage selection provides both robustness and quantifiable advantages over static fusion strategies.

## Introduction

Autonomous vehicles (AVs) are poised to revolutionize transportation, promising significant improvements in road safety, reductions in traffic congestion, and enhanced mobility for diverse user groups^1,2^. Collision avoidance in autonomous driving unfolds through key stages enabled by robust 3D perception: (1) detection and localization of surrounding objects^3^, (2) interpretation of the scene context^4^, and (3) informed real time navigation decisions to avoid hazards and ensure safe trajectory planning^5,6^. This task becomes particularly challenging in complex, dynamic, and unpredictable environments, demanding consistently reliable perception under varying lighting, weather, and traffic conditions.

**Fig. 1:**
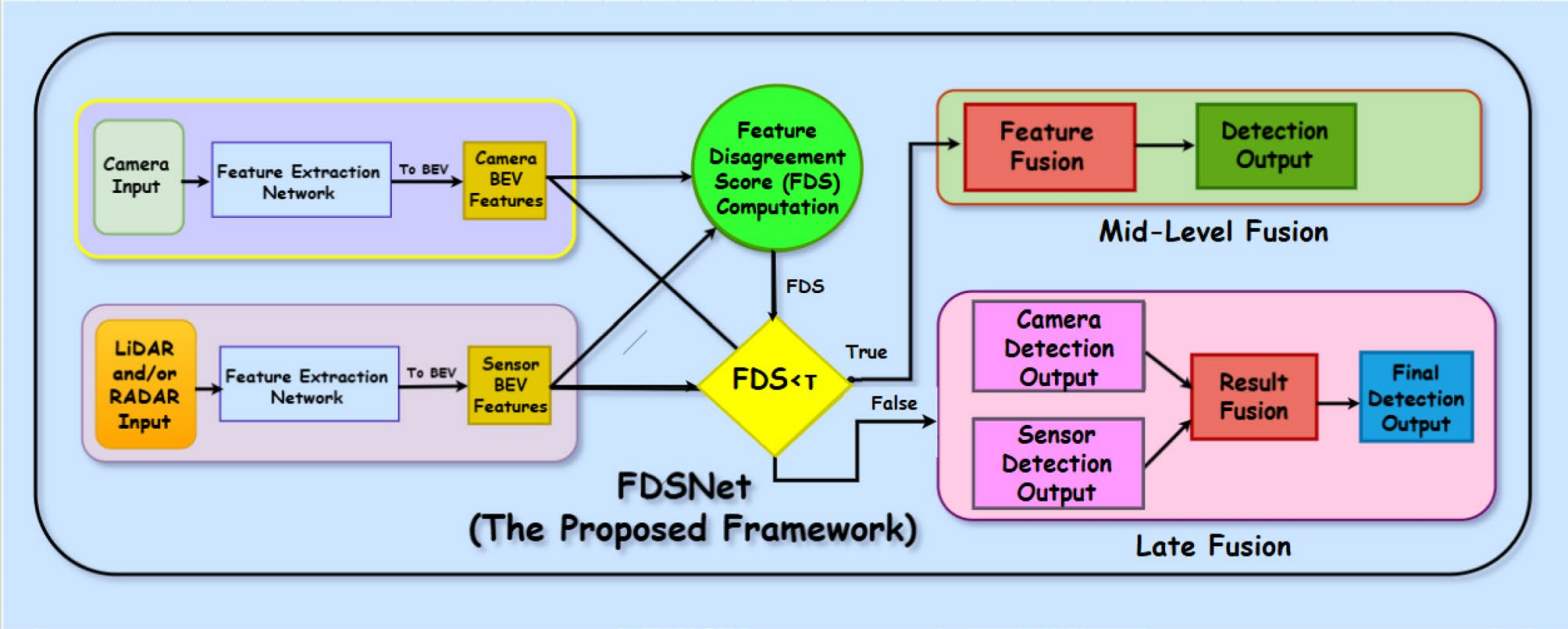
Overview of the proposed FDSNet framework for adaptive sensor fusion in 3D object detection. The framework computes a Feature Disagreement Score (FDS) based on BEV features from Camera and LiDAR/Radar branches. Depending on the FDS value, the system dynamically selects between mid-level (feature fusion) and late-level (result fusion) strategies, enabling robust performance across varying sensor reliability and environmental conditions.

Relying on a single sensor modality for 3D perception often falls short of delivering the necessary robustness and consistency. Each sensor type presents inherent limitations due to its physical sensing principles. Cameras offer detailed texture and color information but remain highly susceptible to lighting variations, performing poorly under low visibility, glare, or nighttime conditions^7–10^. LiDAR sensors provide accurate geometric and distance measurements but struggle with adverse weather conditions, in addition to their significant power demands and cost implications^11,12^. In contrast, Radar demonstrates greater resilience to environmental disturbances and supports long range sensing but is hindered by limited angular resolution and sparse point cloud data, restricting fine grained object detection and classification^13,14^.

To overcome these individual sensor limitations, multi-sensor fusion has emerged as a foundational approach within modern AV perception systems. By integrating complementary information from different modalities such as Radar and Camera^15,16^, LiDAR and Camera^17,18^, or all three Camera, Radar, and LiDAR^19,20^, fusion frameworks construct richer and more resilient representations of the driving environment. This method improves detection accuracy, redundancy, and fault tolerance in real-world scenarios. Nonetheless, most existing fusion frameworks implement a static fusion strategy (early, mid, or late), uniformly applied regardless of scenario dynamics. Such rigid fusion designs neglect variability in cross-sensor consistency, which can fluctuate significantly due to environmental factors like sensor misalignment, occlusion, or varying illumination conditions. For instance, early fusion combining raw or low-level data may inadvertently propagate sensor misalignments and noise throughout the system, reducing overall detection quality^21^. Mid-level fusion tends to exhibit stronger semantic consistency but still assumes that sensor features are consistently aligned^22^. Conversely, late fusion integrates at the decision stage, offering robustness to sensor noise but incurring higher computational overhead and latency, which is a critical drawback in real time AV systems^23^.

To mitigate these trade-offs, recent studies have explored hybrid fusion architectures, merging early-to-mid or mid-to-late fusion combinations^24–26^. These architectures capitalize on early-stage efficiency under optimal sensor alignment and switch to robust, later-stage fusion when inconsistencies arise. However, multi-path hybrid approaches tend to be computationally inefficient, as they execute all fusion branches simultaneously, regardless of necessity. This not only results in substantial memory usage and processing latency, hindering real-time AV applications, but also fails to account for the variability of real-world driving conditions. These challenges become especially critical in adverse scenarios such as heavy rain, fog, or nighttime driving, where camera perception deteriorates, or when LiDAR measurements are disrupted by snow and dust. Likewise, sensor occlusion caused by large vehicles or roadside infrastructure can lead to incomplete observations. Static fusion strategies, which rigidly apply a fixed fusion stage, are unable to adapt to such dynamic inconsistencies, often propagating noise or incurring unnecessary computation. This underscores the necessity of an adaptive fusion mechanism capable of adjusting to varying sensor reliability in real time. In parallel, advances in lightweight convolutional neural networks (CNNs) have highlighted the importance of designing architectures that achieve high accuracy while minimizing computational complexity. Such approaches have been applied successfully in diverse domains, including instrument indication recognition^27^, ancient mural element detection^28^, and biometric verification^29^. These studies demonstrate that carefully designed compact architectures can deliver robust performance under constrained resources, a principle directly aligned with the requirements of autonomous driving perception. Motivated by this trend, our proposed FDSNet extends efficiency-oriented design concepts to multimodal fusion, ensuring both robustness and computational efficiency in real-time scenarios.

Addressing these limitations, we propose FDSNet (Feature Disagreement Score Network), a dynamic fusion framework that adaptively selects the optimal fusion stage either mid-level or late level, based on semantic consistency across sensor modalities. Unlike prior approaches that rigidly execute multiple fusion stages or rely on fixed fusion pipelines, FDSNet introduces a Feature Disagreement Score (FDS) computed at the Bird’s Eye View (BEV) level to quantify semantic inconsistencies between modalities. A global threshold applied to the FDS determines the fusion strategy dynamically, mid-level fusion is activated when semantic consistency is high, enhancing computational efficiency, whereas late-level fusion is triggered under significant disagreement, ensuring robustness. This conditional approach retains the advantages and flexibility of multi-stage fusion methods while significantly reducing redundant computation, making it particularly suited for real time autonomous driving applications. An overview of the FDSNet conditional switching mechanism is illustrated in Fig. 1.

### Our key contributions are summarized as follows

- We propose a dynamic fusion architecture that adaptively switches between mid-level and late-level fusion based on real-time semantic consistency among sensor modalities.
- We introduce the Feature Disagreement Score (FDS), a novel metric that quantifies semantic misalignment at the BEV level and guides conditional fusion decisions.
- We conduct comprehensive experiments on the nuScenes dataset^30^, evaluating three sensor configurations: LiDAR + Camera, Radar + Camera, and LiDAR + Radar + Camera. FDSNet achieves competitive 3D object detection accuracy while significantly reducing computational cost compared to static and hybrid fusion strategies.

## Related work

**Fig. 2:**
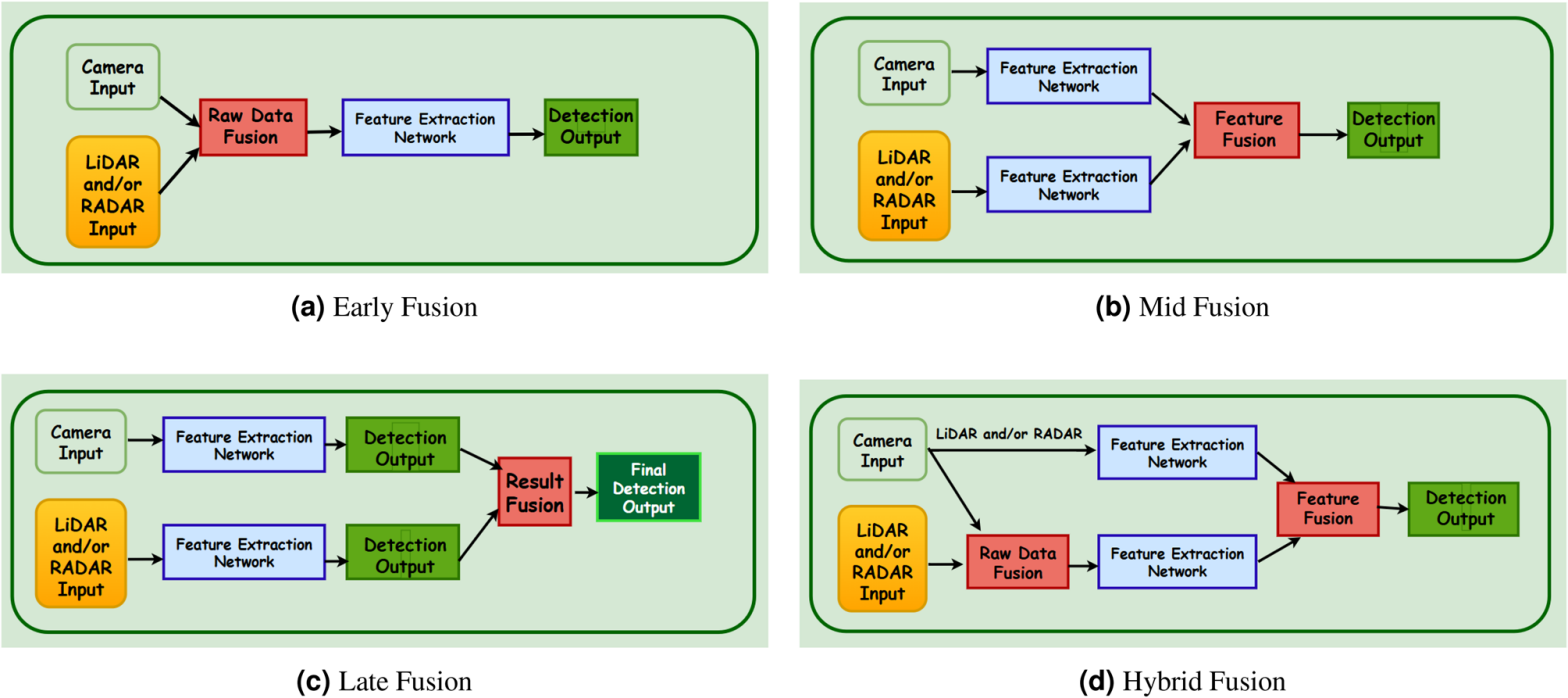
Illustration of different sensor fusion strategies: Early, Mid, Late, and Hybrid fusion schemes.

Multimodal fusion strategies are generally categorized into four main approaches: early, mid-level, late, and hybrid fusion. Each method provides unique trade-offs in terms of computational efficiency, semantic consistency, and adaptability. Fig. 2 illustrates the fundamental stages of these fusion strategies. This section reviews prominent methods in each category, highlighting their strengths and limitations.

### Early fusion

Early fusion combines multimodal sensor data at the input or raw feature stage, prior to independent feature extraction. This fusion strategy leverages the complementarity among different sensor modalities to generate richer input features but faces inherent limitations in computational efficiency and adaptability. A representative example is the ** Multimodal Early Fusion with Attention (MEFA)** model proposed by Dupas et al.^31^. MEFA employs local and global attention modules to fuse visible, infrared, and LiDAR data into a single coherent representation at the raw data level. Specifically, the local attention mechanism captures spatially adjacent feature interactions within each modality, while the global attention mechanism facilitates cross-modal information propagation, enhancing semantic coherence across the fused data. This sophisticated hierarchical attention architecture significantly improves robustness against challenging environmental conditions, such as adverse weather or varying illumination scenarios. However, the computational complexity introduced by these multi-scale attention mechanisms results in substantial computational overhead, limiting the model’s practicality for real time deployment in autonomous systems. Another prominent early fusion method is the **Virtual Sparse Convolution (VirConv)** framework presented by Wu et al.^32^. VirConv projects multi-view LiDAR point clouds and camera images into a common sparse voxel grid representation within a BEV. This voxel based representation enables efficient 3D object detection by leveraging sparse convolutional neural networks, achieving high processing speeds and detection accuracy in well-structured, predictable scenarios. Nonetheless, VirConv’s fixed projection and fusion stages reduce the model’s flexibility, particularly in dynamically changing or semantically inconsistent environments. Its rigid design lacks the capability to adapt fusion parameters based on the evolving semantic relationships among sensor inputs, resulting in potential degradation of accuracy and robustness under complex or unstructured conditions. To overcome these limitations, our proposed FDSNet introduces an adaptive fusion approach guided by a FDS, dynamically selecting between mid-level and late stage fusion based on semantic consistency. This mitigates the performance degradation and computational overhead observed in traditional early fusion methods.

### Mid-level fusion

Mid-level fusion is a modality integration strategy wherein sensor specific features are independently extracted prior to their fusion, preserving the distinct semantic and structural properties intrinsic to each modality. Unlike early fusion, mid-level fusion capitalizes on modality specific representations, enabling more effective fusion after extracting specialized features from each sensor independently. This approach is particularly advantageous in scenarios where direct integration at the raw data level may obscure critical domain specific information. **BEVFusion4D**^33^exemplifies this approach. BEVFusion4D first independently extracts BEV feature representations from LiDAR and camera inputs. Subsequently, it leverages a LiDAR-guided view transformation mechanism to accurately project and spatially align image features within a unified BEV coordinate system. The aligned features are then fused using spatial and temporal fusion modules designed to incorporate both spatial contextual coherence and temporal consistency. Although it efficiently resolves issues related to cross modal misalignment and effectively captures temporal dynamics, its fusion process operates unconditionally irrespective of the semantic coherence or agreement among the modalities. Consequently, noise or erroneous data from one modality may propagate into the fused representation, incurring unnecessary computational overhead and reducing model robustness. Similarly, **DeepStep**^21^, utilizes an incremental step wise fusion strategy. DeepStep progressively merges modality specific 2D image features and 3D LiDAR features via a hierarchical spatiotemporal transformer architecture. This step wise fusion paradigm enhances contextual reasoning and improves semantic understanding by gradually refining multi modal feature representations. Despite these benefits, it also exhibits limitations, particularly in its absence of conditional control mechanisms. It consistently applies identical fusion processes regardless of semantic disagreements, potentially degrading overall fusion effectiveness when certain modalities are noisy, inaccurate, or semantically inconsistent. In the context of Radar and Camera fusion, **CRN**^34^ presents a mid-level approach that effectively integrates high level modality specific features through a dynamic spatial fusion strategy. By leveraging Radar’s motion stability and Camera’s semantic richness, CRN improves robustness against visual degradation and sparsity. However, similar to other mid-level methods, it lacks an explicit mechanism for selectively gating or adjusting the fusion process based on semantic agreement, which may limit its adaptability in inconsistent or noisy environments. Addressing these limitations, FDSNet employs the FDS metric to dynamically select the optimal fusion stage. By retaining mid-level fusion only when cross-modal alignment is strong and shifting to late fusion in cases of significant feature disagreement, FDSNet significantly reduces redundant computation and enhances robustness.

### Late fusion

Late fusion independently processes each sensor modality through separate, dedicated feature extraction and decision making pipelines, integrating their outputs only at the final decision making stage. This approach inherently maintains the modularity of processing streams and is particularly robust against scenarios involving sensor degradation or failure, as the fusion is performed at a high level decision or prediction space. Nevertheless, this strategy may fail to fully leverage intermediate semantic cues and complementary information available at earlier stages of processing, potentially limiting overall fusion performance. The **C-CLOCs** framework^35^, employs a contrastive learning based approach designed to align object level predictions from LiDAR and camera modalities. It accomplishes this by performing confidence calibration and Intersection-over-Union (IoU) based matching of object proposals generated independently by each modality. By aligning predictions post-hoc, it effectively reduces false positives and enhances consistency across sensor modalities. Despite these advantages, C-CLOCs is fundamentally constrained by its reliance on fixed, post-hoc alignment techniques. As a consequence, it lacks the flexibility to dynamically adapt its fusion strategy based on real time fluctuations or variations in modality specific performance, potentially compromising performance under rapidly changing environmental conditions or varying sensor reliability. Another model is **BAFusion**, introduced by Chen et al.^17^, utilizes a bidirectional attention mechanism to perform modality specific late stage fusion. It independently generates predictions from LiDAR and camera inputs, subsequently employing bidirectional cross attention modules to integrate these high level modality specific predictions. This approach effectively captures high level semantic correlations and retains modularity, enabling ease of sensor specific adaptation and maintenance. However, similar to other late fusion strategies, BAFusion applies fusion unconditionally, without explicitly evaluating or quantifying the level of cross modal semantic alignment or disagreement. Consequently, in challenging scenarios characterized by conflicting or semantically misaligned sensor outputs, unconditional fusion may degrade overall detection and classification accuracy. To effectively mitigate these limitations and exploit the inherent strengths of both mid-level and late stage fusion strategies, in contrast our proposed FDSNet dynamically quantifies the degree of semantic disagreement across modalities in real time. Unlike previous late fusion methods, FDSNet selectively applies late fusion only when significant disagreement exists, otherwise favoring computationally efficient mid-level fusion. Thus, it combines the robustness benefits of late fusion with adaptive computational efficiency.

### Dynamic and hybrid fusion

Recent advances in multi modal fusion for autonomous driving have prompted the emergence of dynamic and hybrid fusion methods, which integrate sensor data across multiple abstraction levels such as (point-level, mid-level, decision-level). These hybrid approaches aim to combine the strengths of individual fusion strategies, achieving a balance between robustness, flexibility, and computational efficiency by adaptively utilizing complementary information at varying processing stages. **DecoratingFusion**^25^ exemplifies a hybrid approach, enriching LiDAR point clouds with image aligned features at the input stage and subsequently refining them using mid-level BEV-based cross modal attention. Similarly, **MS-Occ**^26^ proposes a multi-stage LiDAR–Camera fusion architecture, combining mid-level projection of 2D image features into 3D voxel spaces with late stage semantic aggregation from multiple viewpoints. Although effective in improving geometric and semantic understanding, these hybrid methods execute all fusion stages unconditionally, resulting in increased latency and computational costs even when simpler fusion strategies might suffice. **RCBEV**^36^ further contributes to this direction by addressing the spatial misalignment between Radar and Camera modalities through modality specific feature adaptation and alignment modules, improving the reliability of Radar-Camera fusion in BEV-based 3D detection. Although effective in improving geometric and semantic understanding, these hybrid methods execute all fusion stages unconditionally, resulting in increased latency and computational costs even when simpler fusion strategies might suffice. In addition, they lack an explicit mechanism to evaluate or quantify semantic consistency across modalities, meaning that fusion is applied regardless of whether sensor features are aligned or conflicting. This inability to adapt often propagates redundant or noisy computations. Conditional fusion approaches, like **HydraFusion**^37^, offer a promising direction by selecting among predefined fusion branches based on scene context or learned contextual features. it employs a context-aware gating mechanism, dynamically choosing fusion paths guided by external metadata (e.g., weather) or learned context. However, each selected branch still follows a rigid fusion pipeline, performing fusion irrespective of real time semantic alignment, thus risking redundant computation and suboptimal performance.

To address these shortcomings comprehensively, the proposed FDSNet utilizes a real time computed FDS to dynamically select either mid-level or late fusion stages based explicitly on semantic consistency avoiding the redundant execution of all fusion branches and directly resolving the limitations of existing hybrid strategies. This conditional fusion approach further enhances computational efficiency, and maintains robust perception tailored explicitly for real time autonomous driving applications. Table 1 summarizes the reviewed sensor fusion approaches, categorized by fusion stage, and highlights their core limitations regarding adaptability, efficiency, and semantic consistency.

**Table 1:** Sensor fusion models with their strengths and limitations.

Stage	Model	Strengths	Limitations
Early	MEFA^31^	High-resolution fusion, fine-grained spatial feature alignment	Rigid fusion, high computational cost, limited real-time use
	VirConv^32^	Lightweight, efficient voxel feature propagation	Limited adaptability, struggles with semantic inconsistencies
Mid-Level	BEVFusion4D^33^	Temporal BEV fusion, consistent scene aggregation	Executes fusion regardless of feature alignment, redundant computations
	CRN^34^	adar-Camera feature fusion with dynamic spatial reasoning	Lacks semantic consistency gating , sensitive to modality disagreement
	DeepStep^21^	Progressive integration, improved temporal continuity	Unconditional fusion execution, limited adaptability
Late	C-CLOCs^35^	Modality-specific decision making, robustness to misalignment	Post-hoc alignment, limited semantic feature utilization
	BAFusion^17^	Uncertainty-aware fusion, reliability weighting	Unconditional fusion, limited adaptability to sensor disagreement
Hybrid	DecoratingFusion^25^	Multi-stage refinement, dense feature propagation	Executes all fusion stages unconditionally, increased latency
	MS-Occ^26^	Multi-scale occupancy reasoning, strong spatial coverage	Performs all fusion stages unconditionally, computationally intensive
	HydraFusion^37^	Flexible per-branch processing, adaptable fusion routes	Static fusion pipelines within each branch, unnecessary computations
	RCBEV^36^	Radar-Camera feature alignment, modality-specific spatial adaptation	Limited to Radar-Camera pairs, lacks generalized modality fusion

**Algorithm 1:**
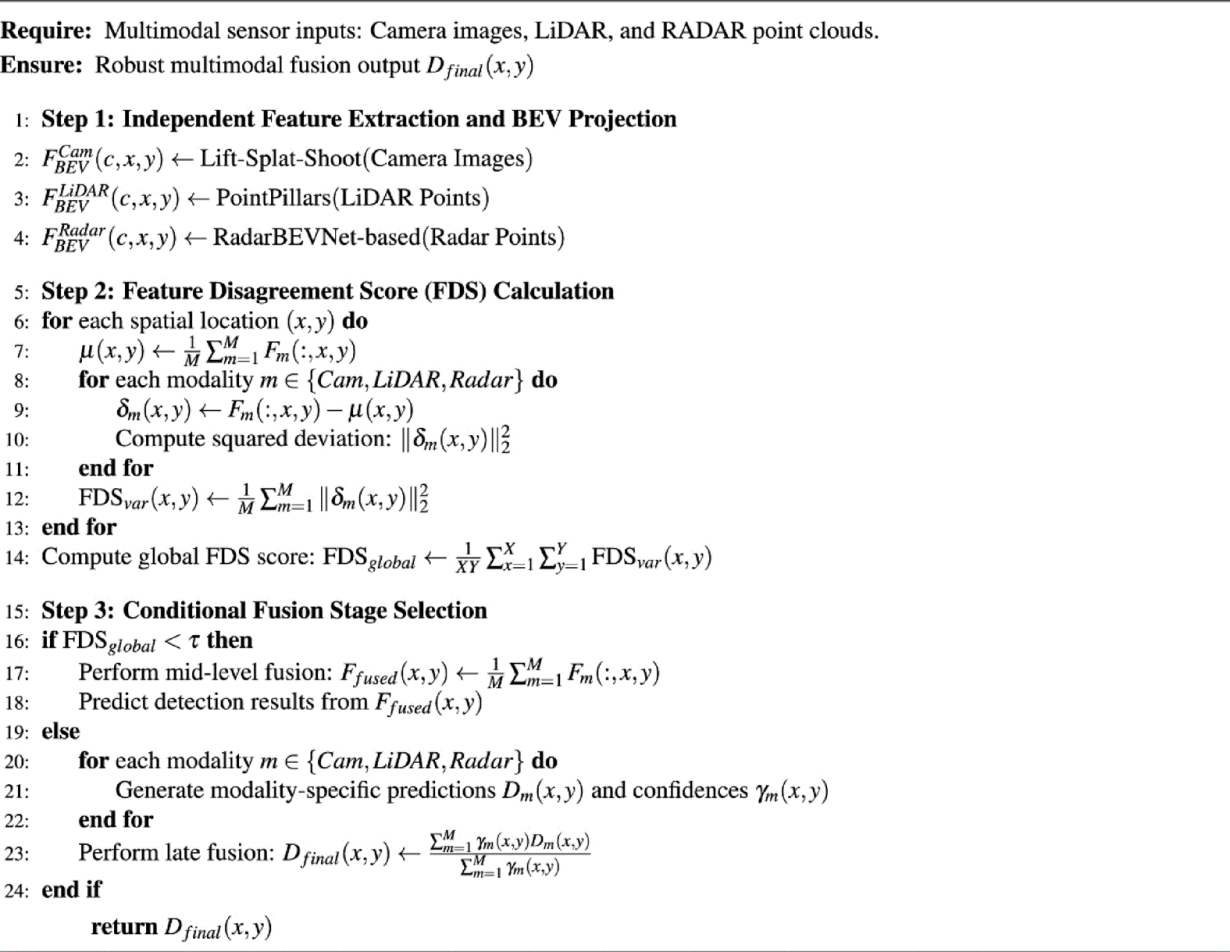
Proposed FDSNet fusion Algorithm.

## Proposed work

The proposed FDSNet architecture dynamically integrates multimodal sensor data by conditionally selecting optimal fusion stage based on real-time semantic alignment across the three sensor configurations: Camera+LiDAR (C+L), Camera+Radar (C+R), and Camera+LiDAR+Radar (C+L+R). This algorithm operates in three primary steps: (1) Independent feature extraction and spatial alignment, wherein each sensor modality is processed independently and mapped into a unified Bird’s Eye View (BEV) representation. (2) Computation of the Feature Disagreement Score (FDS), a variance-based metric quantifying semantic alignment and disagreement between modalities. and (3) Conditional fusion stage selection, dynamically choosing between mid-level feature fusion and late-stage decision fusion based on the global FDS value. This adaptive mechanism enhances computational efficiency and robustness for real-time autonomous driving applications. Early-stage raw feature fusion was intentionally excluded, as it tends to amplify noise, increase dimensionality, and propagate modality-specific errors, making it less effective for reliable real-time decision-making. The pseudocode describing the overall algorithm is provided in Algorithm 1.

### Feature extraction from multi-modal sensors

To extract semantically meaningful representations from heterogeneous sensor data, we adopt a three-branch design independently extracting mid-level features from Camera, LiDAR, and RADAR sensors. Each modality-specific representation is subsequently projected into a unified BEV representation to facilitate consistency assessment and spatial alignment. The use of BEV is motivated by its ability to provide compact, spatially coherent, and semantically rich representations–ideal for multi modal fusion. In contrast, voxel-based representations, which often memory intensive and computationally expensive due to their sparse 3D structure^38^. Image plane projections, meanwhile, suffer from geometric distortions and misalignment across modalities, as direct projection into camera viewpoints can warp spatial relationships and degrade 3D accuracy^33^. By projecting into BEV, we avoid these pitfalls while preserving both geometric precision and semantic density. Thus, BEV offers a robust fusion foundation compared to alternative fusion schemes.

#### Camera stream

To generate BEV features from multi-view camera images, we adopt the Lift-Splat-Shoot (LSS) mechanism^39^. Compared to transformer based view transformation methods like BEVFormer^40^ or PETR^41^, which offer strong performance but come at a significantly higher computational cost, LSS offers a strong balance between accuracy and real time performance due to its simplified architecture and reduced latency. The process begins by the Lift step, in this step image features $f \in \mathbb {R}^{C \times H \times W}$, (where *C* is the number of feature channels, *H* and *W* are the spatial dimensions) are extracted using a backbone ResNet^42^ and followed by FPN neck^43^ module to enhance spatial detail and contextual representation. A depth classifier then predicts a discrete depth distribution $\alpha \in \mathbb {R}^D$ for each pixel, where *D* is the number of sampled depth bins. Each pixel at image coordinates (*u*, *v*) is lifted into a set of 3D frustum points (*x*, *y*, *z*) using the camera intrinsic *K* and extrinsic $[R \mid t]$. Here, $K\in \mathbb {R}^{3 \times 3}$ encodes the camera’s internal parameters (focal lengths and principal point), while $R \in \mathbb {R}^{3 \times 3}$ is a rotation matrix that orients the camera in 3D space, and $t \in \mathbb {R}^{3 \times 1}$ is a translation vector specifying the camera’s position in the world coordinate system. The full projection to 3D is given in (1), following the standard pinhole camera model and coordinate transformation defined in^44^:

1 $$\begin{aligned} \begin{bmatrix} x \\ y \\ z \end{bmatrix} = R^\top \left( \alpha _d \cdot K^{-1} \begin{bmatrix} u \\ v \\ 1 \end{bmatrix} - t \right) \end{aligned}$$

where $\alpha _d$ scales each 3D point according to depth probability. In the Splat step, the lifted 3D points from all views are aggregated into a voxel grid *V*(*c*, *x*, *y*, *z*). To obtain the final BEV feature map, a vertical pooling operation is applied along the height (z) axis while preserving the channel dimension:

2 $$\begin{aligned} F_{\text {BEV}}^{\text {Cam}}(c, x, y) = \sum _z V(c, x, y, z) \end{aligned}$$

**Fig. 3:**
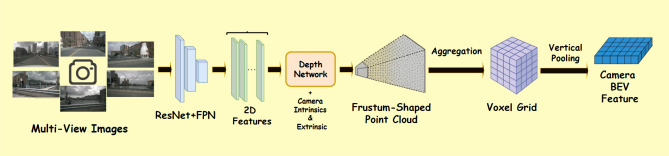
BEV feature generation from multi-view camera inputs. 6 surround view images are first processed using a ResNet+FPN backbone to extract 2D image features. A depth network lifts each pixel into a 3D frustum using camera intrinsics and extrinsics. The lifted points are then aggregated into a voxel grid and vertically pooled to produce the final BEV representation.

This BEV representation encodes both semantic and geometric information and serves as the unified spatial format for downstream fusion assessment. A visual illustration of the full camera-to-BEV transformation pipeline is provided in Fig. 3.

#### LiDAR stream

For the LiDAR We adopt the PointPillars framework^45^ to efficiently convert LiDAR point clouds into BEV features. Compared to voxel-based encoders like VoxelNet^46^ or sparse 3D convolutional neural network (CNN) models such as SECOND^47^, PointPillars eliminates the need for expensive 3D convolutions by operating in the 2D BEV plane, achieving a favorable trade-off between accuracy and speed as reported in the prior benchmarks^48^. The input point cloud $P \in \mathbb {R}^{N \times D}$, where *N* is the number of points and *D* includes spatial coordinates and intensity values, is first discretized into vertical columns (pillars) across the XY plane. Each non-empty pillar is encoded using PointNet^49^ to produce a fixed length feature vector. These features are then scattered into a 2D pseudo-image, preserving spatial structure. Finally, the pseudo-image is processed by a 2D CNN to produce the final BEV feature map:

3 $$\begin{aligned} F_{\text {BEV}}^{\text {LiDAR}} \in \mathbb {R}^{C \times X \times Y} \end{aligned}$$

By capturing local geometry and intensity, this representation facilitates spatial alignment essential for computing the FDS across sensor modalities.

#### RADAR stream

To efficiently extract BEV features from radar point clouds in real time, we employ a streamlined point-based backbone inspired by RadarBEVNet^50^. In contrast to alternative radar processing approaches such as heatmap-based representations^34^, which suffer from limited geometric precision and low spatial resolution, the point-based method provides a more spatially accurate and lightweight feature encoding strategy suitable for sparse radar data. The input data is represented as $R \in \mathbb {R}^{K \times 5}$ where *K* is the number of radar points in a frame, and each point is represented by a 5-dimensional vector comprising its 3D spatial coordinates (*x*, *y*, *z*) and Doppler-compensated velocity components $(v_x, v_y)$. Each point is encoded using a simplified PointNet^49^ architecture, which applies a shared Multi-Layer Perceptron (MLP) to project raw inputs into a higher dimensional feature space, followed by a global max pooling operation to capture contextual information. The radar feature encoding is given by:

4 $$\begin{aligned} f = \text {Concat}[\text {MLP}(f), \text {MaxPool}(\text {MLP}(f))] \end{aligned}$$

The encoded features are then scattered into a structured 2D grid using RCS-aware scattering, which spreads each point’s influence over multiple BEV locations according to its Radar Cross Section (RCS). The resulting BEV feature map:

5 $$\begin{aligned} F_{\text {BEV}}^{\text {Radar}} \in \mathbb {R}^{C \times X \times Y} \end{aligned}$$

serves as a spatially aligned representation used in downstream fusion and FDS computation.

### FDS calculation

To quantify modality disagreement over spatially aligned BEV features, we adopt a variance-based FDS that captures semantic inconsistencies across the Camera, LiDAR, and Radar streams. Each modality-specific BEV feature map is denoted as $F_m \in \mathbb {R}^{C \times X \times Y}$, where $F_m \in \{F_\text {BEV}^{\text {Cam}}, F_\text {BEV}^{\text {LiDAR}}, F_\text {BEV}^{\text {Radar}}\}$, as introduced in Equations (2), (3), and (5), respectively. From each BEV map, the local feature value at spatial coordinate (*x*, *y*) and channel *c* for modality *m* is denoted as $f_m^{(c)}(x, y) \in \mathbb {R}$. The average channel-wise feature at each location is computed using the following formulation:

6 $$\begin{aligned} \mu ^{(c)}(x, y) = \frac{1}{M} \sum _{m=1}^{M} f_m^{(c)}(x, y), \quad \text {for } c = 1, \dots , C \end{aligned}$$

Here, $M \in \{1, 2, 3\}$ represents the number of modalities (Camera, LiDAR, Radar) participating in the current fusion instance. Next, we determine each modality deviation from the mean feature vector calculated in Eq. (7) as:

7 $$\begin{aligned} \delta _m^{(c)}(x, y) = f_m^{(c)}(x, y) - \mu ^{(c)}(x, y), \quad \text {for } c = 1, \ldots , C \end{aligned}$$

The level of disagreement per modality at each location is then computed by the squared L2-norm of these deviations, as defined in Eq. (8):

8 $$\begin{aligned} \Vert \delta _m(x, y)\Vert _2^2 = \sum _{c=1}^{C} \left( \delta _m^{(c)}(x, y) \right) ^2 \end{aligned}$$

This formulation quantifies the extent to which modality *m* deviates from the average representation across all modalities at a specific spatial location. A higher value indicates greater disagreement or inconsistency in the feature encoding of that modality relative to the others. Finally, the overall **FDS** at location $(x, y)$ is computed by averaging the squared deviations derived in Eq. (8) across all sensor modalities, as defined in Eq. (9):

9 $$\begin{aligned} \text {FDS}_{\text {var}}(x, y) = \frac{1}{M} \sum _{m=1}^{M} \Vert \delta _m(x, y)\Vert _2^2 \end{aligned}$$

This metric quantifies the consistency of feature representations across sensors at each spatial location and enables downstream tasks to identify ambiguous or uncertain regions. To extend this to the scene level, we define **Global Feature Disagreement Score** (${\textbf {FDS}}_{{\textbf {global}}}$) by spatially averaging local values across the entire BEV grid:

10 $$\begin{aligned} \text {FDS}_{\text {global}} = \frac{1}{X \cdot Y} \sum _{x=1}^{X} \sum _{y=1}^{Y} \text {FDS}_{\text {var}}(x, y) \end{aligned}$$

This scalar reflects the overall semantic consistency across all modalities. Local noise or isolated disagreements are smoothed, while systematic cross-modal misalignments remain emphasized, making global FDS a robust metric for guiding adaptive fusion stage selection.

### Conditional fusion stage selection

Building upon the calculated $\text {FDS}_{\text {global}}$, we propose a dynamic fusion control strategy that conditionally selects between mid-fusion and late fusion stages. This approach leverages the global agreement across spatially aligned BEV features to determine whether to fuse at the feature level for confident, consistent scenes or defer to decision-level fusion for ambiguous, uncertain regions. A low $\text {FDS}_{\text {global}}$ indicates strong modal agreement, while a high value suggests potential semantic misalignment or sensor disagreement. We then apply a conditional rule to select the fusion stage *S* based on a experimentally tunable threshold $\tau$ as:

11 $$\begin{aligned} S = {\left\{ \begin{array}{ll} \mathscr {F}_{\text {mid}}, & \text {if } \text {FDS}_{\text {global}} < \tau \\ \mathscr {F}_{\text {late}}, & \text {otherwise} \end{array}\right. } \end{aligned}$$

**Fig. 4:**
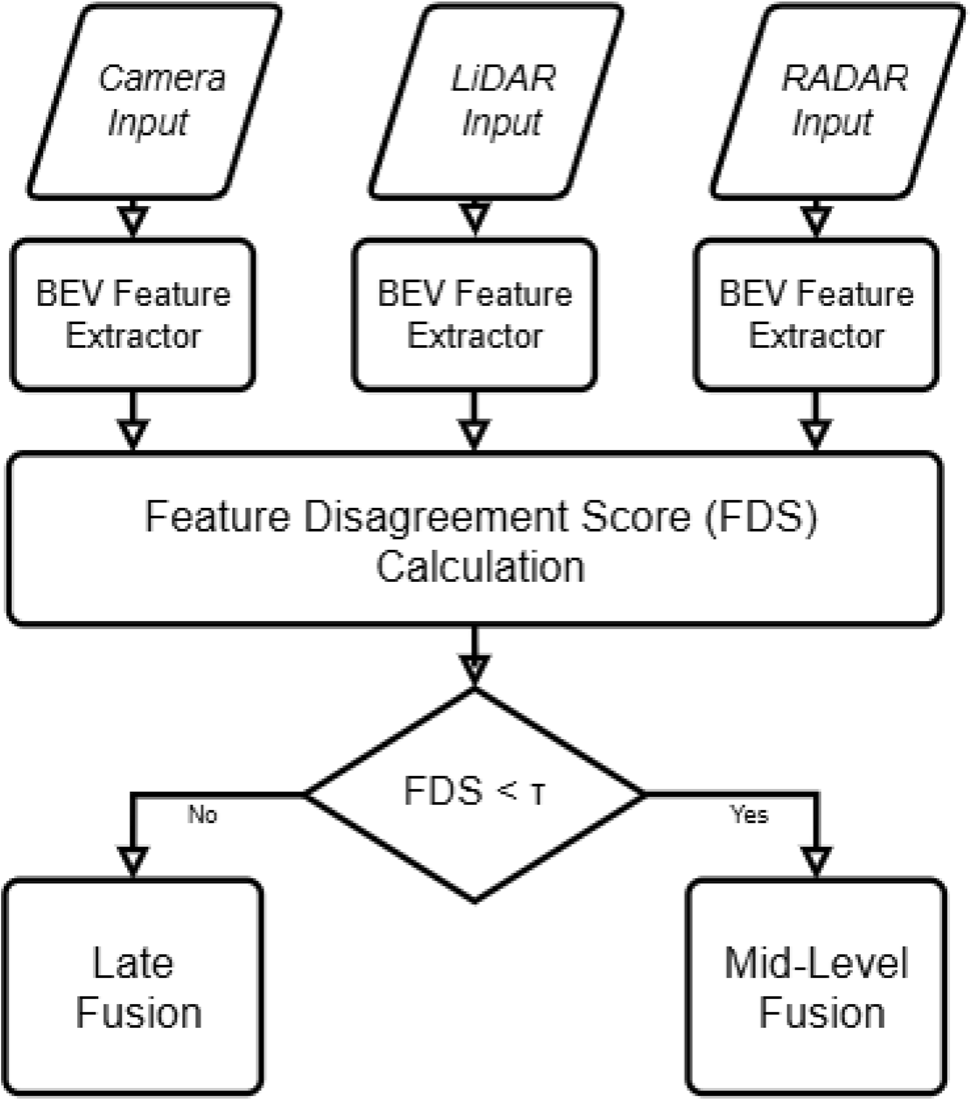
Flowchart of FDSNet’s conditional fusion, where the FDS threshold $\tau$ determines mid-level or late fusion selection.

The overall conditional switching process is illustrated in Fig. 4, where BEV features from Camera, LiDAR, and Radar are evaluated through the FDS and compared against a threshold $\tau$ to select the appropriate fusion stage. In the mid-fusion stage, denoted by $\mathscr {F}_{\text {mid}}$, spatially aligned BEV features from all available modalities are combined through an element-wise average:

12 $$\begin{aligned} F_{\text {fused}}(x, y) = \frac{1}{M} \sum _{m=1}^{M} f_m(x, y) \end{aligned}$$

This simple yet effective fusion strategy assumes equal trust across modalities in regions where the FDS indicates strong semantic consistency. Compared to more complex fusion mechanisms such as attention-based fusion, or convolutional encoders as used in BEVFusion^51^, this approach introduces minimal computational overhead and is highly suitable for real time deployment. By avoiding additional parameters $\mathscr {F}_{\text {mid}}$ provides a fast, deterministic alternative while still benefiting from the redundancy and complementarity of multi-modal inputs under low disagreement conditions. A detailed comparison of this method against alternative mid-fusion strategies is provided in Table 2.

In contrast to mid-level fusion, late fusion operates at the decision level, where each modality: Camera, LiDAR, and Radar independently generates complete detection outputs, including class probability scores and corresponding bounding box parameters at each BEV location $(x, y)$. Each modality $m$ provides a prediction $D_m(x, y)$ alongside a confidence estimate $\gamma _m(x, y) \in [0, 1]$, indicating its reliability at that position. The final fused decision $D_{\text {final}}(x, y)$ is computed using a confidence-weighted aggregation:

13 $$\begin{aligned} D_{\text {final}}(x, y) = \frac{ \sum _{m=1}^{M} \gamma _m(x, y) \cdot D_m(x, y) }{ \sum _{m=1}^{M} \gamma _m(x, y) } \end{aligned}$$

this approach ensures that predictions from more reliable modalities dominate the final outcome, while those from less certain sources are down weighted. Compared to simple averaging or rule based voting, the confidence weighted fusion mechanism offers a more adaptive and fine grained strategy, dynamically reflecting sensor reliability at each spatial location. This makes it particularly effective under adverse conditions, such as sensor occlusion, misalignment, or environmental interference, thereby enhancing the robustness and reliability of multi-sensor object detection in real time driving environments. A detailed comparison of this method with other representative late-stage fusion approaches is presented in Table 3. In addition, for clarity, we provide a summary table of the key terms and symbols used in FDSNet, which consolidates the mathematical notations introduced in Table 4.

**Table 2:** Comparison of mid-level fusion strategies.

Fusion method	Fusion operation	Complexity	Real-time suitability	Parameter overhead
Element-wise Average^52^	Sum over spatially aligned BEV features	Low	High	Low
Attention-based Fusion^34^	Local and global attention over concatenated features	High	Low	High
Convolutional Fusion^51^	Concatenation followed by convolutional encoding in BEV space	Medium	Medium	Medium

**Table 3:** Comparison of late fusion strategies.

Fusion method	Fusion operation	Complexity	Real-time suitability	Parameter overhead
Confidence-Weighted Fusion^35^	Weighted sum of predictions using modality-wise confidence scores:	Medium	High	Low
Simple Averaging^53^	Uniform average of outputs without confidence weights	Low	Medium	None
Rule-Based Voting^54^	Majority or priority-based fusion of class labels or boxes	Low	Medium	None
Learned Fusion Gate^55^	Task-specific gating network trained to select or weight predictions from each modality	High	Low	High

**Table 4:** Summary of symbols and terms in FDSNet.

Symbol/term	Definition	Role in FDSNet
$\textbf{F}_{m} \in \mathbb {R}^{C \times X \times Y}$	Modality-specific BEV feature map (Camera, LiDAR, Radar)	Input feature representation per modality
$f_{m}^{(c)}(x,y)$	Feature value at channel *c*, location (*x*, *y*), modality *m*	Local spatial feature element
$\mu ^{(c)}(x,y)$	Mean feature across modalities at (*x*, *y*), Eq. (6)	Baseline for measuring disagreement
$\delta _{m}^{(c)}(x,y)$	Deviation of modality *m* from mean at (*x*, *y*), Eq. (7)	Captures modality-specific differences
$\Vert \delta _{m}(x,y)\Vert _{2}^{2}$	Squared L2-norm of deviations, Eq. (8)	Quantifies per-modality disagreement
$\textrm{FDS}_{\textrm{var}}(x,y)$	Average disagreement across modalities at (*x*, *y*), Eq. (9)	Local Feature Disagreement Score
$\textrm{FDS}_{\textrm{global}}$	Spatial average of $\textrm{FDS}_{\textrm{var}}(x,y)$, Eq. (10)	Global consistency metric for fusion stage decision
$\tau$	Threshold parameter, Eq. (11)	Controls mid- vs late-fusion switching
$F_{\textrm{fused}}$	Mid-level fusion output, Eq. (12)	Element-wise average when agreement is high
$D_{m}(x,y), \ \gamma _{m}(x,y)$	Detection output and confidence for modality *m*, Eq. (13)	Inputs for late fusion
$D_{\textrm{final}}(x,y)$	Final decision after confidence-weighted fusion, Eq. (13)	Robust fused output under uncertainty

## Experiments

In this section, we begin by presenting the benchmark dataset employed to evaluate the proposed FDSNet. We then describe the experimental settings and implementation details.

### Dataset

**Fig. 5:**
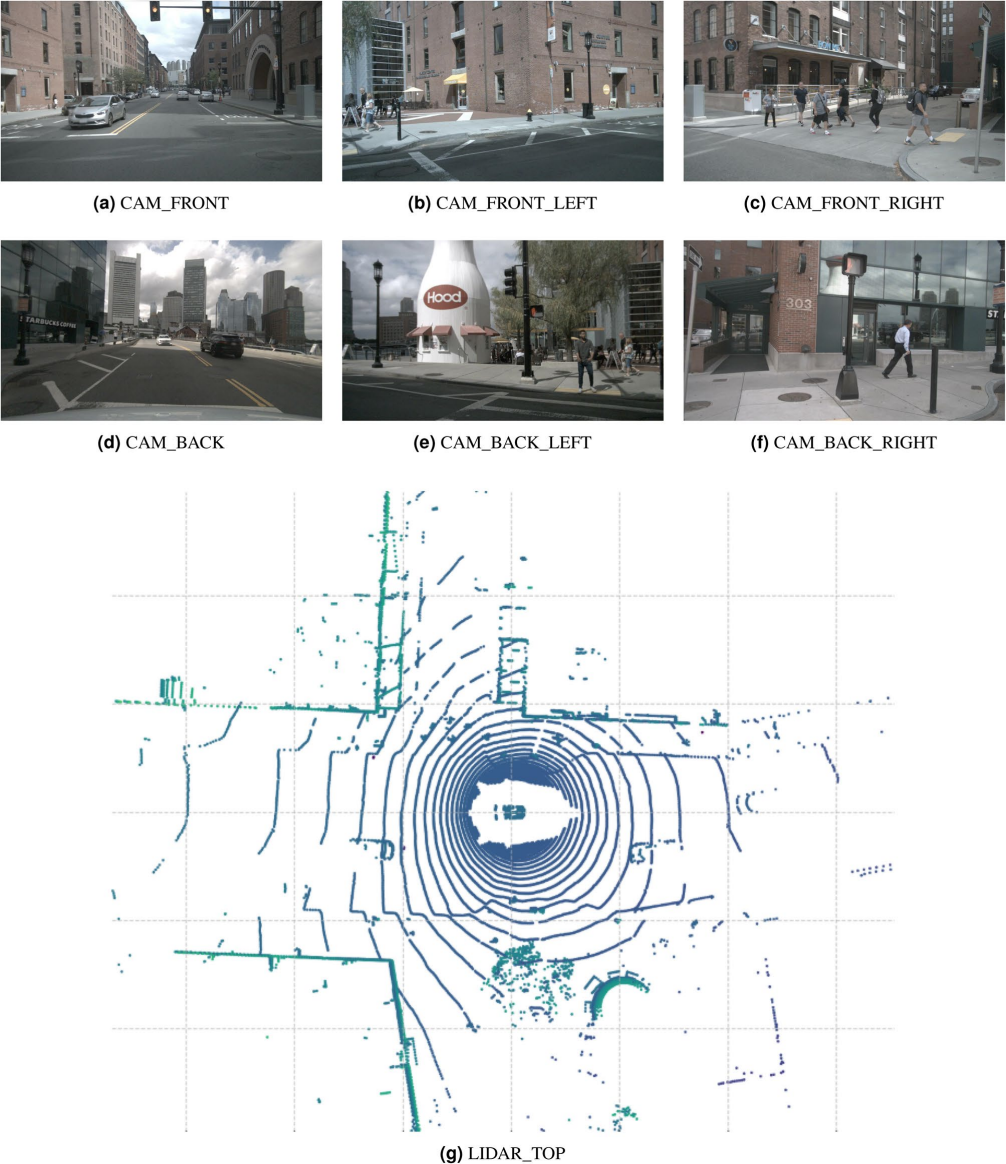
Representative samples from the nuScenes Dataset^30^.

We conduct our experiments on the nuScenes dataset^30^, a large scale benchmark for autonomous driving perception tasks. It provides synchronized multi-sensor data, including six surround view cameras, five RADAR sensors, and one 32-beam LiDAR scanner, captured at 2 Hz across diverse urban scenarios in Boston and Singapore. Each scene offers full 360° coverage and is annotated with 3D bounding boxes for 10 object categories, including cars, pedestrians, trucks, and bicycles. A representative samples from the nuScenes dataset are provided in Fig. 5, including RGB images from six camera views (top two rows) and a LiDAR top view (bottom), to visualize the spatial coverage and complementary characteristics of these two sensor modalities.

### Experimental setup and implementation details

#### Computational environment

All experiments were performed on a high performance computing workstation equipped with an Intel Core i7 CPU running at 3.40 GHz, 64 GB of system memory, and two NVIDIA GeForce GTX 1080 Ti GPU. The proposed framework was implemented in PyTorch^56^ using open-sourced MMDetection3D^57^, which provides modular support for 3D object detection and multi-sensor fusion tasks.

#### Implementation details

The proposed FDSNet includes three modality specific backbone networks,a ResNet^42^ followed by FPN neck^43^ module for camera input initialized from ImageNet^58^ pretrained weights, a PointPillars backbone for LiDAR(trained from scratch following Lang et al.^45^), and a streamlined RadarBEVNet inspired point-based backbone for RADAR data (trained from scratch with He initialization as in Zhou et al.^50^). Training was structured into three sequential stages to ensure robust feature learning and stable cross-modal convergence: First, the LiDAR backbone was trained individually for 20 epochs to extract robust 3D representations from point clouds. Second, the camera backbone was independently trained for 12 epochs utilizing standard augmentations (random flipping with 0.5 probability, rotation ±0.3925 rad, and scaling [0.95, 1.05]). Finally, the multi-sensor fusion network was trained jointly for an additional 18 epochs, integrating Camera, LiDAR, and RADAR features into a unified BEV representation, with parameters from camera and LiDAR backbones frozen as recommended by prior fusion studies^59,60^. For evaluation, we split the nuScenes dataset, which comprises roughly 1000 scenes, into 700 for training, 150 for validation, and 150 for testing, ensuring no overlap across subsets, following the official nuScenes^30^. All training stages employed the same optimization setup. AdamW^61^ with an initial learning rate of $1 \times 10^{-3}$, weight decay of 0.01, gradient clipping at norm 35, linear warm-up (1000 iterations), and multi-step decay at epochs 14 and 17 by a factor of 0.1,with a batch size of 4 samples per GPU (on 2 GPUs). Point cloud data from LiDAR and RADAR was voxelized into pillars of size $0.25\,\text {m} \times 0.25\,\text {m} \times 8\,\text {m}$, covering $[-50\,\text {m}, 50\,\text {m}]$ in the X/Y axes and $[-5\,\text {m}, 3\,\text {m}]$ in the Z-axis. For the threshold parameter $\tau$, we experimentally evaluated values in the range [0.3–0.7] on the validation set and selected $\tau$ = 0.5 as the default setting, since it provided the best balance between mid- and late-stage fusion. This regimen ensured effective modality specific learning, enhanced robustness, computational efficiency, and reproducibility.

## Evaluation metrics

To rigorously quantify the performance of the proposed FDSNet for 3D object detection, we adopt the official nuScenes detection evaluation protocol^30^, which offers a multifaceted assessment across spatial localization, object orientation, scale estimation, velocity prediction, and attribute classification. The evaluation focuses on a comprehensive set of metrics, including the *mean Average Precision* (mAP), *mean Average Translation Error* (mATE), *mean Average Scale Error* (mASE), *mean Average Orientation Error* (mAOE), *mean Average Velocity Error* (mAVE), *mean Average Attribute Error* (mAAE), and the overall *nuScenes Detection Score* (NDS). These metrics collectively provide a holistic view of a model’s capability to accurately detect and characterize objects in 3D space, reflecting not only detection precision but also geometric fidelity and semantic richness.

### Mean average precision (mAP)

In alignment with the nuScenes evaluation protocol, mean Average Precision is computed using a center-distance-based matching approach rather than the traditional IoU, reducing sensitivity to object size and orientation. A detection is considered a true positive if its 2D center lies within a threshold distance $d \in D = \{0.5, 1, 2, 4\}$ meters from the ground-truth box center. For each class $c \in C$ and each threshold $d \in D$, the Average Precision $\text {AP}_{c,d}$ is defined as the area under the precision-recall curve, excluding operating points where precision or recall falls below 10%. The final mean Average Precision (mAP) is then computed as the average across all classes and thresholds:

14 $$\begin{aligned} \text {mAP} = \frac{1}{|C||D|} \sum _{c \in C} \sum _{d \in D} \text {AP}_{c,d} \end{aligned}$$

### Mean average scale error (mASE)

The mean Average Scale Error (mASE) quantifies the deviation in object dimensions between predicted and ground truth 3D bounding boxes. Unlike traditional *IoU* metrics, mASE isolates scale discrepancies by first aligning the predicted box with the ground truth in terms of translation and orientation, so that only scale contributes to the residual error. It is formally defined as:

15 $$\begin{aligned} \text {ASE} = 1 - \text {IoU}(\hat{B}_{\text {pred}}, B_{\text {gt}}) \end{aligned}$$

Where $\hat{B}_{\text {pred}}$ denotes the rescaled predicted box aligned with the ground truth box $B_{\text {gt}}$ in both position and yaw orientation.

### Mean average translation error (mATE)

This metric measures the average Euclidean distance (in meters) between predicted and ground truth bounding box centers:

16 $$\begin{aligned} \text {mATE} = \frac{1}{N} \sum _{i=1}^{N} \Vert \textbf{t}_i^{\text {pred}} - \textbf{t}_i^{\text {gt}} \Vert _2 \end{aligned}$$

### Mean average orientation error (mAOE)

The mean Average Orientation Error (mAOE) quantifies the accuracy of predicted object orientations by measuring the smallest angular difference between the predicted yaw angle $\theta _{\text {pred}}$ and the ground truth yaw angle $\theta _{\text {gt}}$, expressed in radians. This error is computed over a full $360^\circ$ period for most object classes, except for symmetric objects such as barriers, where a $180^\circ$ period is used. The orientation error for each matched prediction is defined as:

17 $$\begin{aligned} \text {AOE} = \min \left( \left| \theta _{\text {pred}} - \theta _{\text {gt}} \right| ,\, 2\pi - \left| \theta _{\text {pred}} - \theta _{\text {gt}} \right| \right) \end{aligned}$$

The final mAOE is obtained by averaging the AOE values across all valid classes and matched detections, providing a robust measure of directional estimation performance that is independent of translation or scale.

### Mean average velocity error (mAVE)

The mean Average Velocity Error (mAVE) evaluates the accuracy of predicted object motion by calculating the L2 norm (Euclidean distance) between the predicted and ground truth velocity vectors in the 2D plane, expressed in meters per second (m/s). This metric captures discrepancies in both magnitude and direction of motion and is defined for each matched detection as:

18 $$\begin{aligned} \text {AVE} = \left\| \textbf{v}_{\text {pred}} - \textbf{v}_{\text {gt}} \right\| _2 \end{aligned}$$

where $\textbf{v}_{\text {pred}}$ and $\textbf{v}_{\text {gt}}$ represent the predicted and ground truth velocity vectors, respectively. The final mAVE is computed by averaging AVE values across all matched predictions and valid object classes that exhibit motion.

### Mean average attribute error (mAAE)

The mean Average Attribute Error (mAAE) quantifies the accuracy of attribute prediction by computing the complement of classification accuracy. Specifically, the per sample attribute error is defined as:

19 $$\begin{aligned} \text {AAE} = 1 - \text {acc} \end{aligned}$$

where **acc** denotes the proportion of correctly predicted attributes over the total number of valid attribute annotations. The final mAAE is obtained by averaging AAE values across all matched detections and applicable object classes (excluding classes like cones or barriers where attributes are undefined).

### nuScenes detection score (NDS)

The nuScenes Detection Score (NDS) provides a unified metric to evaluate both detection accuracy and the quality of 3D bounding box estimation. It combines the mAP with five True Positive (TP) error metrics translation (mATE), scale (mASE), orientation (mAOE), velocity (mAVE), and attribute classification (mAAE) into a single score. These TP metrics, denoted collectively as mTP, capture the estimation fidelity of critical object properties. The NDS is computed as:

20 $$\begin{aligned} \text {NDS} = \frac{1}{10} \left( 5 \cdot \text {mAP} + \sum _{\text {mTP}} \left( 1 - \min \left( 1, \text {mTP}\right) \right) \right) \end{aligned}$$

Here, the summation over mTP refers to the set $\{\text {mATE}, \text {mASE}, \text {mAOE}, \text {mAVE}, \text {mAAE}\}$. Each metric is clipped to the [0, 1] range to ensure stability and comparability. The final score balances classification performance (via mAP) with the regression accuracy of object properties.

## Results and discussion

**Fig. 6:**
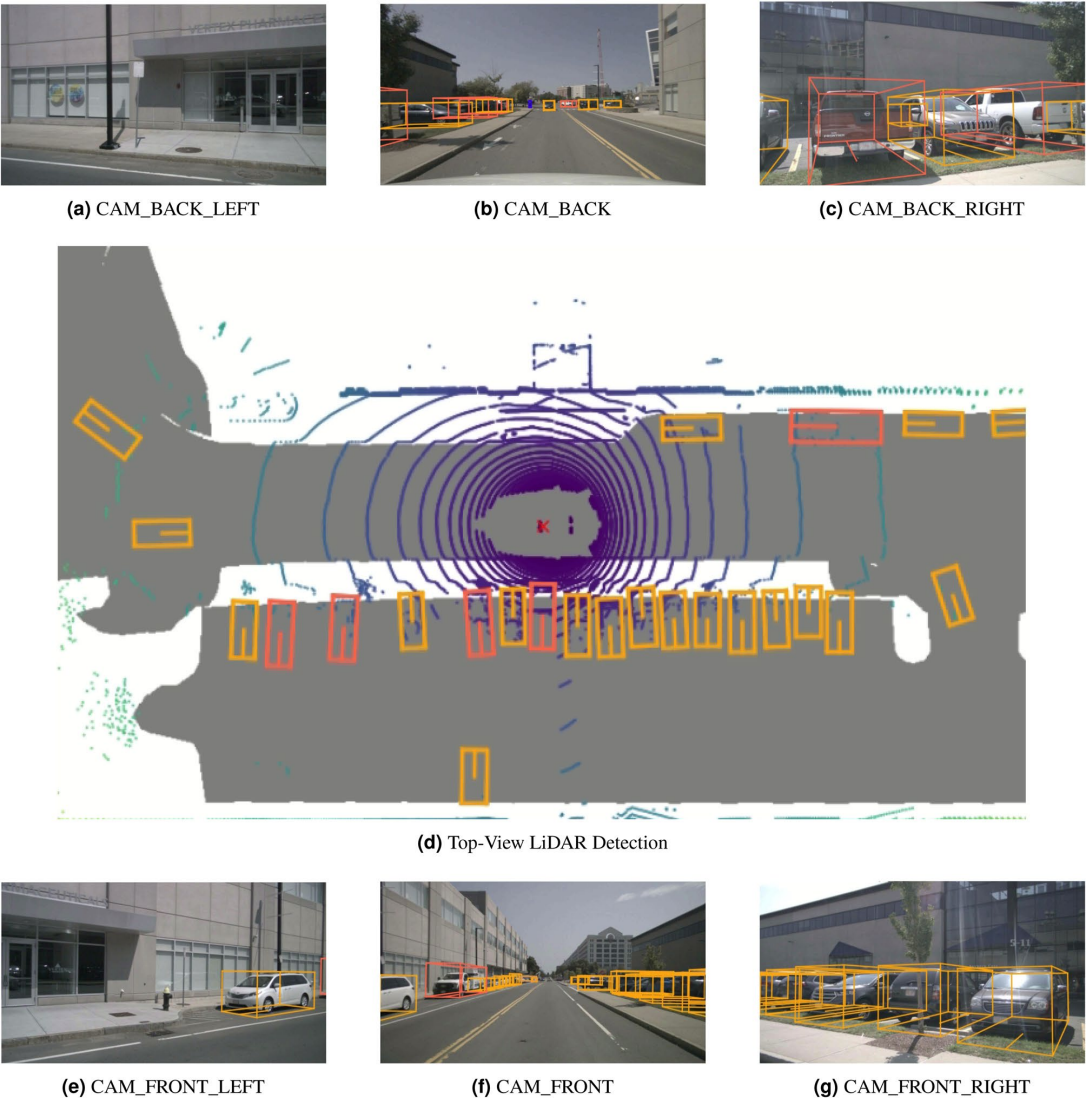
Detection results from our proposed **FDSNet** model. The fused multi-camera, LiDAR, and Radar views showcase the effectiveness of our adaptive fusion strategy for robust 3D object detection under diverse perspectives.

**Fig. 7:**
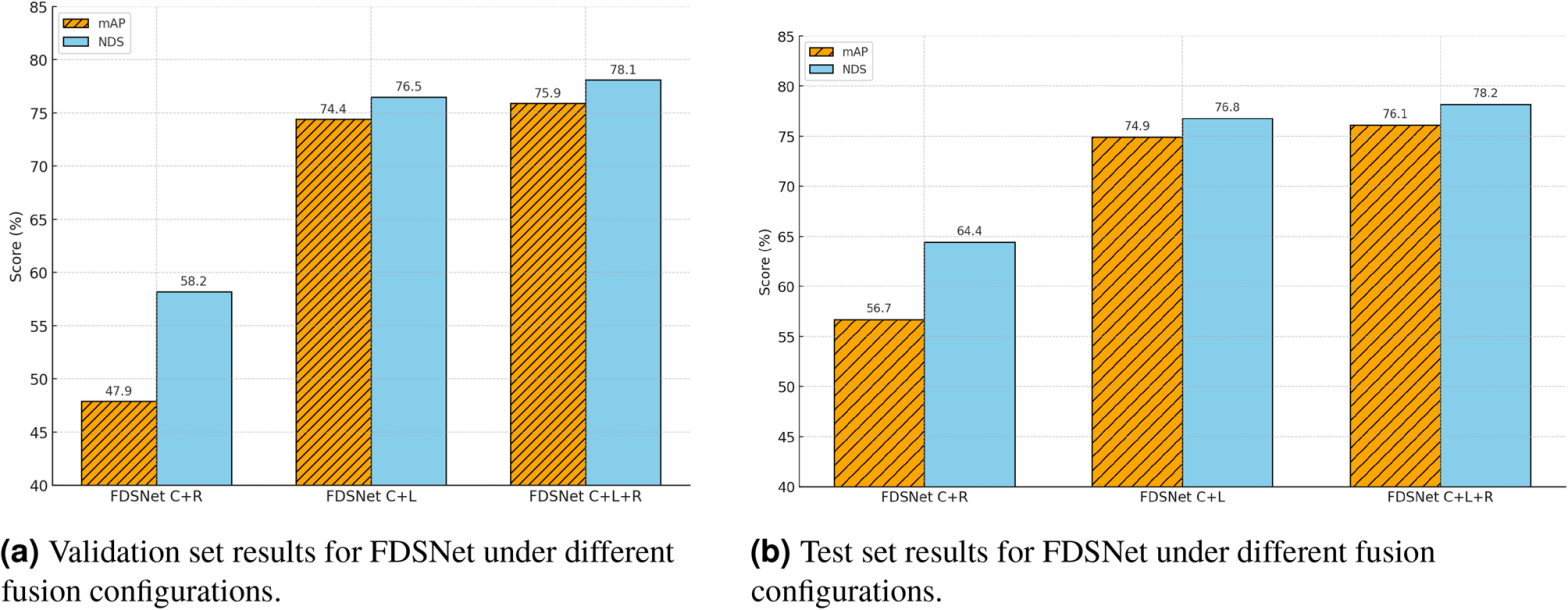
Performance comparison of mAP and NDS for different sensor fusion configurations using FDSNet on the nuScenes dataset.

All experiments were conducted on the nuScenes benchmark^30^, where we evaluated our proposed FDSNet framework under three sensor fusion configurations: Camera+RADAR (C+R), Camera+LiDAR (C+L), and Camera+LiDAR+RADAR (C+L+R). As summarized in Table 5 and Table 6, the complete fusion configuration (C+L+R) achieved the highest performance across both the validation set (75.9% mAP, 78.1% NDS) and test set (76.1% mAP, 78.2% NDS), outperforming recent state-of-the-art multi-modal detectors such as PolarFusion and IS-Fusion. This superior performance arises from the complementary strengths of the three modalities. The Camera provides dense semantic and texture information, LiDAR contributes precise geometric depth and structural cues, while Radar offers robustness under adverse conditions such as rain, fog, or poor illumination, together with FDSNet’s adaptive fusion strategy, which aligns the fusion stage with the level of cross-modal consistency. The (C+L) configuration also showed competitive performance (74.4% mAP, 76.5% NDS on validation, 74.9% mAP, 76.8% NDS on test), exceeding PolarFusion and IS-Fusion by noticeable margins and reinforcing the effectiveness of integrating high-resolution geometry with visual context. Despite RADAR’s sparsity and noisier returns, the (C+R) setting yielded promising results (56.7% mAP, 64.4% NDS on test), outperforming top radar fusion baselines such as RCBEVDet and CRN. This indicates that even in sparse modalities, our adaptive fusion mechanism effectively suppresses cross-modal inconsistencies and enhances robustness. Qualitative results are shown in Fig. 6. A detailed breakdown of mAP and NDS under each fusion configuration is illustrated in Fig. 7.

**Table 5:** Quantitative comparison of 3D object detection performance on the nuScenes validation set. ‘C’, ‘R’, and ‘L’ denote input from Camera, Radar, and LiDAR sensors, respectively.

Method	Input	NDS$\uparrow$	mAP$\uparrow$	mATE$\downarrow$	mASE$\downarrow$	mAOE$\downarrow$	mAVE$\downarrow$	mAAE$\downarrow$
CenterFusion^62^	C+R	45.3	33.2	0.649	0.263	0.535	0.540	0.142
CRAFT^63^	C+R	51.7	41.1	0.494	0.276	0.454	0.486	0.176
RCBEVDet^16^	C+R	56.3	45.3	0.492	0.269	0.449	0.230	0.188
RCBEV4D^36^	C+R	49.7	38.1	0.526	0.272	0.445	0.465	0.185
CRN^34^	C+R	54.3	44.8	0.518	0.283	0.552	0.279	0.180
CR3DT^64^	C+R	45.6	35.1	-	-	-	0.47	-
BEVDet^65^	C	39.2	31.2	0.691	0.272	0.523	0.909	0.247
BEVDepth^66^	C	47.5	35.1	0.639	0.267	0.479	0.428	0.198
SOLOFusion^67^	C	53.4	42.7	0.567	0.274	0.411	0.252	0.188
StreamPETR^68^	C	54.0	43.2	0.581	0.272	0.413	0.295	0.195
RCBEVDet^16^	C+R	56.8	45.3	0.486	0.285	0.404	0.220	0.192
PolarFusion^69^	C+L	75.1	73.3	-	-	-	-	-
IS-Fusion^70^	C+L	74.0	72.8	-	-	-	-	-
ProFusion3D^71^	C+L	73.6	71.1	-	-	-	-	-
FDSNet (Ours)	C+R	58.2	47.9	0.468	0.251	0.319	0.270	0.140
FDSNet (Ours)	C+L	76.5	74.4	0.398	0.228	0.288	0.240	0.110
FDSNet (Ours)	**C+L+R**	**78.1**	**75.9**	**0.385**	**0.219**	**0.275**	**0.229**	**0.105**

**Table 6:** Quantitative comparison of 3D object detection performance on the nuScenes test set. ‘C’, ‘R’, and ‘L’ denote input from Camera, Radar, and LiDAR sensors, respectively.

Method	Input	NDS$\uparrow$	mAP$\uparrow$	mATE$\downarrow$	mASE$\downarrow$	mAOE$\downarrow$	mAVE$\downarrow$	mAAE$\downarrow$
KPConvPillars^72^	R	13.9	4.9	0.823	0.428	0.607	2.081	1.000
CenterFusion^62^	C+R	44.9	32.6	0.631	0.261	0.516	0.614	0.115
RCBEV^36^	C+R	48.6	40.6	0.484	0.257	0.587	0.702	0.140
MVFusion^73^	C+R	51.7	45.3	0.569	0.246	0.379	0.781	0.128
CRAFT^63^	C+R	52.3	41.1	0.467	0.268	0.456	0.519	0.114
BEVFormer^40^	C	56.9	48.1	0.582	0.256	0.375	0.378	0.126
PETRv2^41^	C	58.2	49.0	0.561	0.243	0.361	0.343	0.120
BEVDepth^66^	C	60.5	51.5	0.446	0.242	0.377	0.324	0.135
SOLOFusion^67^	C	61.9	54.0	0.453	0.257	0.376	0.276	0.148
CRN^34^	C+R	62.4	57.5	0.416	0.264	0.456	0.365	0.130
SparseBEV^74^	C	63.6	55.6	0.485	0.244	0.332	0.246	0.117
StreamPETR^68^	C	63.6	55.0	0.493	0.241	0.343	0.243	0.123
RCBEVDet^16^	C+R	63.9	55.0	0.390	0.234	0.362	0.259	0.113
PolarFusion^69^	C+L	76.1	74.5	-	-	-	-	-
IS-Fusion^70^	C+L	75.2	73.0	-	-	-	-	-
FDSNet (Ours)	C+R	64.4	56.7	0.402	0.236	0.341	0.248	0.114
FDSNet (Ours)	C+L	76.8	74.9	0.384	0.227	0.323	0.233	0.108
FDSNet (Ours)	**C+L+R**	**78.2**	**76.1**	**0.371**	**0.222**	**0.315**	**0.225**	**0.105**

## Ablation and efficiency analysis

To comprehensively evaluate the adaptability and efficiency of FDSNet, we conducted an extensive ablation study covering four aspects: (1) the effect of the threshold parameter $\tau$ on adaptive stage selection, (2) the impact of fusion strategy and dynamic switching, (3) computational efficiency across different sensor configurations, and (4) category-wise detection performance. Together, these analyses validate the effectiveness of the proposed FDS in guiding real-time fusion decisions, improving perception accuracy under varying sensor agreements, and maintaining consistent detection quality across object classes.

### Effect of threshold $\tau$

**Fig. 8:**
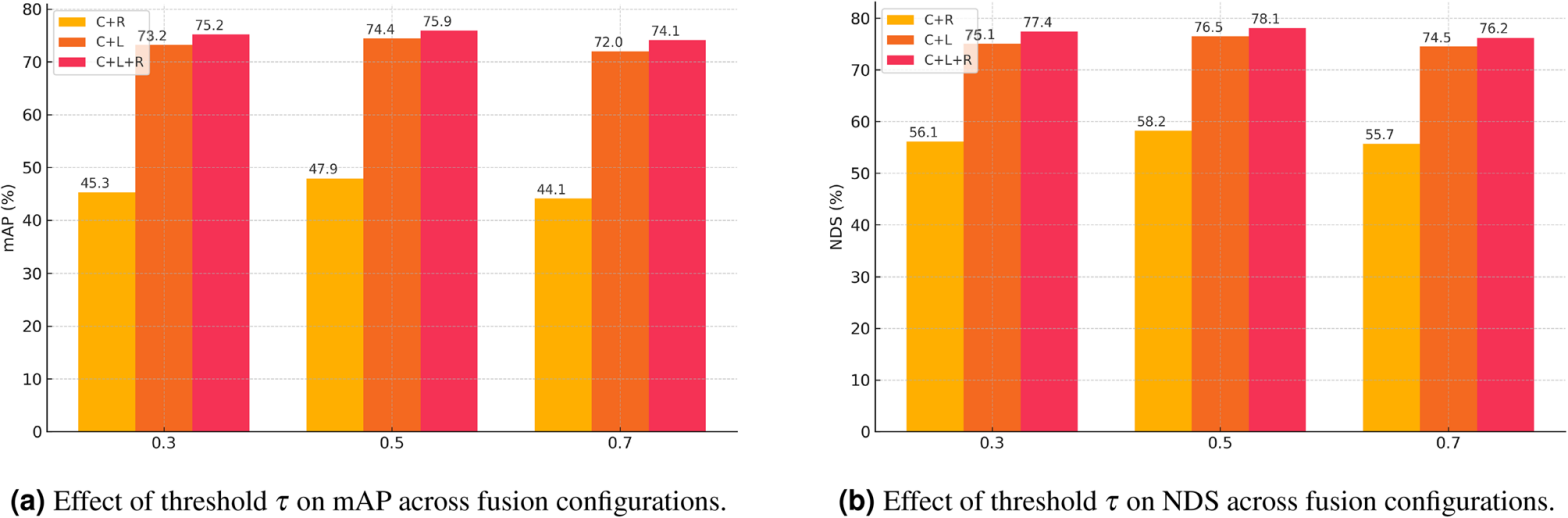
Performance variation with respect to fusion selection threshold $\tau$ in FDSNet.

We investigate the influence of the threshold parameter $\tau$, which governs the balance between mid and late stage fusion across configurations in Table 7. As expected, a lower $\tau$ favors late fusion by making it harder to satisfy the condition $FDS_\text {global} < \tau$, while a higher $\tau$ biases toward mid fusion. For example, $\tau = 0.3$ leads to late fusion dominance, while $\tau = 0.7$ shifts the preference to mid fusion, with $\tau = 0.5$ providing the best trade-off. The performance variation associated with threshold tuning is visualized in Fig. 8, which plots the effect of $\tau$ on both mAP and NDS across all sensor configurations. Together, these results validate that FDSNet’s dynamic fusion mechanism adapts effectively to sensor agreement levels and consistently improves performance while preserving computational efficiency.

**Table 7:** Effect of the FDS threshold $\tau$ on detection performance across different sensor configurations. A lower threshold biases the system toward late fusion by making it harder to satisfy $\text {FDS}_{\text {global}} < \tau$, while a higher threshold increases mid-fusion dominance. Balanced performance is observed near $\tau = 0.5$.

Threshold $\varvec{\tau }$	Sensor configuration	Fusion stage bias	mAP $\uparrow$	NDS $\uparrow$
0.3	C + R	Late-Fusion Dominant	45.3	56.1
0.5	C + R	Balanced	**47.9**	**58.2**
0.7	C + R	Mid-Fusion Dominant	46.2	57.0
0.3	C + L	Late-Fusion Dominant	72.2	75.1
0.5	C + L	Balanced	**74.4**	**76.5**
0.7	C + L	Mid-Fusion Dominant	73.3	75.9
0.3	C + L + R	Late-Fusion Dominant	74.2	77.1
0.5	C + L + R	Balanced	**75.9**	**78.1**
0.7	C + L + R	Mid-Fusion Dominant	74.8	77.3

### Ablation on fusion strategy

The ablation results in Table 8 show that incorporating dynamic switching via the Feature Disagreement Score (FDS) yields consistent improvements across all evaluation metrics compared with static fusion strategies using all three modalities (Camera, LiDAR, and Radar). In the fixed mid-fusion setup, features from the three sensors are merged before detection, effectively combining the semantic richness of the camera with the geometric precision of LiDAR and the motion awareness of radar. However, this approach assumes perfect spatial alignment between modalities, but in practice, small calibration drift or the inherent sparsity of radar measurements can lead to feature misalignment, which in turn degrades orientation and velocity estimation (mAOE = 0.289, mAVE = 0.252), limiting overall accuracy (mAP = 73.2, NDS = 75.4). The fixed late-fusion configuration, which merges independent detections at the decision level, enhances robustness to sensor noise through confidence weighting, LiDAR primarily governs localization accuracy (mATE = 0.395) while Radar refines motion estimation (mAVE = 0.238), but lacks intermediate feature interaction, resulting in suboptimal semantic integration (mAP = 74.3, NDS = 76.5). In contrast, dynamic FDSNet adaptively switches between mid and late fusion according to real-time semantic consistency, using mid-fusion under strong cross-modal agreement and late-fusion when discrepancies arise. This mechanism improves detection across all metrics (mAP = 75.9, NDS = 78.1, mATE = 0.385, mASE = 0.219, mAOE = 0.275, mAVE = 0.229, mAAE = 0.105) by optimally combining the complementary strengths of camera semantics, LiDAR geometry, and radar motion cues, ensuring robust and consistent multimodal perception for autonomous driving.

**Table 8:** Ablation study on fusion strategy and dynamic switching using all three modalities (Camera + LiDAR + Radar) on the nuScenes validation set.

Fusion strategy	Switching mode	NDS $\uparrow$	mAP $\uparrow$	mATE $\downarrow$	mASE $\downarrow$	mAOE $\downarrow$	mAVE $\downarrow$	mAAE $\downarrow$
Mid-Fusion	Fixed	75.4	73.2	0.404	0.226	0.289	0.252	0.118
Late-Fusion	Fixed	76.5	74.3	0.395	0.223	0.281	0.238	0.112
**FDSNet (Ours)**	**Dynamic**	**78.1**	**75.9**	**0.385**	**0.219**	**0.275**	**0.229**	**0.105**

### Computational efficiency analysis

To evaluate the scalability and runtime efficiency of the proposed framework, we analyzed FDSNet’s computational complexity, throughput, and detection accuracy under the three sensor configurations:(C+R),(C+L), and (C+L+R) setup. As summarized in Table 9, FDSNet exhibits a consistent and interpretable trade-off between perception accuracy and computational cost across all modality combinations. The lightweight C+R configuration achieves robust performance (mAP = 47.9, NDS = 58.2) with the lowest computational load (318 GFLOPs, 6.2 GB) and the highest throughput (31 FPS), making it suitable for resource-constrained deployments. The C+L configuration offers a strong balance between precision and efficiency (mAP = 74.4, NDS = 76.5) with moderate requirements (365 GFLOPs, 7.0 GB, 27 FPS). Extending to the full C+L+R setup yields the highest detection accuracy (mAP = 75.9, NDS = 78.1) with only a modest increase in complexity (412 GFLOPs, 7.8 GB) and an effective runtime of 24 FPS

**Table 9:** Computational efficiency, runtime, and accuracy of FDSNet under different sensor configurations on the nuScenes validation set.

FDSNet Configuration	Params (M)	GFLOPs	GPU Memory (GB)	FPS	mAP$\uparrow$	NDS$\uparrow$
C + R	**68.5**	**318**	**6.2**	**31.0**	47.9	58.2
C + L	78.9	365	7.0	27.1	74.4	76.5
C + L + R	82.4	412	7.8	24.3	**75.9**	**78.1**

### Class-level performance analysis

To provide a deeper understanding of category-level detection behavior, Table 10 presents the per-class mAP and NDS of FDSNet across different sensor configurations on the nuScenes validation set. The lightweight (C+R) configuration achieves moderate accuracy overall but exhibits reduced precision for small targets such as pedestrian and bicycle, where the sparse radar returns and limited spatial priors constrain feature alignment. Incorporating (C+L) markedly enhances structural reasoning and object boundary localization through dense geometric cues, resulting in substantial improvements across all categories, particularly for large objects truck, bus and barrier. Extending to the full configuration (C+L+R) yields the highest per-class performance by leveraging radar-derived velocity fields to disambiguate motion states and refine temporal consistency in dynamic scenarios. These results confirm that FDSNet’s adaptive fusion mechanism scales effectively with sensing diversity, integrating complementary modality characteristics to achieve balanced and robust 3D perception across all object classes.

**Table 10:** Per-class 3D detection results (mAP, NDS) for FDSNet under different sensor configurations on the nuScenes validation set.

FDSNet Configuration	Input	Car	Truck	Bus	Trailer	Constr.	Ped.	Motor	Bicycle	Traf.	Barrier	mAP	NDS$\uparrow$
C+R	C+R	81.2	55.3	33.1	61.4	57.9	68.2	51.7	32.8	84.1	77.6	**47.9**	58.2
C+L	C+L	88.5	63.1	38.9	74.6	67.8	79.1	82.5	59.8	89.4	88.7	**74.4**	76.5
C+L+R	**C+L+R**	**89.8**	**67.8**	**44.5**	**77.6**	**68.3**	**81.8**	**85.3**	**65.6**	**93.4**	**91.1**	**75.9**	**78.1**

## Limitations and future work

### Limitations

Despite demonstrating strong performance and adaptability, the proposed FDSNet has some limitations that warrant consideration. First, the effectiveness of the FDS depends on accurate cross-modal alignment within the BEV representation. Calibration drift or asynchronous sensor timing can impair semantic consistency estimation, potentially leading to suboptimal fusion decisions. Second, the fusion threshold parameter $\tau$, which governs mid and late stage switching, is currently selected empirically. While the ablation study confirms stable performance around $\tau =0.5$, automatic or data-driven threshold adaptation would further enhance robustness under diverse conditions. Third, FDSNet performs semantic consistency estimation on a per-frame basis, without explicitly modeling temporal correlations across frames. Incorporating temporal consistency could improve stability in rapidly changing environments or when sensor reliability fluctuates. Finally, although the efficiency analysis shows real-time inference (24–31 FPS), additional optimization will be needed for low-power or embedded deployments where computational resources are limited.

### Future work

Future research directions may address these limitations and enhance the generalization capabilities of the proposed framework. Incorporating advanced calibration aware mechanisms or self-supervised alignment approaches could mitigate sensor misalignment issues, thus improving the reliability of the FDS. Additionally, developing an adaptive and learnable threshold determination mechanism for instance, via reinforcement learning or meta learning strategies could enable automatic and context-aware selection of the fusion stage without extensive manual tuning.

## Conclusion

In this work, we presented FDSNet, a dynamic multimodal fusion framework designed to overcome the limitations of static fusion strategies in autonomous driving. By introducing the FDS, our approach quantifies semantic consistency across Camera, LiDAR, and Radar streams, enabling real-time selection between mid-level and late fusion. This adaptive mechanism ensures that the system maintains both robustness under sensor disagreement and efficiency in favorable conditions. The experiments across multiple sensor configurations demonstrate that FDSNet provides a unified solution that scales seamlessly from sparse to dense modalities. The framework highlights how adaptive stage selection can suppress cross-modal inconsistencies while avoiding unnecessary computation, making it particularly suitable for real-time perception tasks.

## Data Availability

This study relies on the publicly available nuScenes dataset^30^, which can be accessed through the official website at: https://www.nuscenes.org/nuscenes. The dataset is openly accessible and was used in accordance with its respective terms of use. The corresponding author N. M. O should be contacted if someone wants to request the data from this study.
